# Exploring the transparency mechanism and evaluating the effect of public reporting on prescription: a protocol for a cluster randomized controlled trial

**DOI:** 10.1186/s12889-015-1454-6

**Published:** 2015-03-21

**Authors:** Xin Du, Dan Wang, Xuan Wang, Shiru Yang, Xinping Zhang

**Affiliations:** School of Medicine and Health Management, Tongji Medical College, HuaZhong University of Science and Technology, Wuhan, Hubei Province China

**Keywords:** Study protocol, Transparency mechanism, Public reporting, Intervention, Prescription, Cluster randomized controlled trial

## Abstract

**Background:**

The public reporting of health outcomes has become one of the most popular topics and is accepted as a quality improvement method in the healthcare field. However, little research has been conducted on the transparency mechanism, and results are mixed with regard to the evaluation of the effect of public reporting on quality improvement. The objectives of this trial are to investigate the transparency mechanism and to evaluate the effect of public reporting on prescription at the level of individual participants.

**Methods/Design:**

This study involves a cluster randomized controlled trial conducted in 20 primary-care facilities (clusters). Eligible clusters are those facilities with excellent hospital information systems and that have agreed to participate in the trial. The 20 clusters are matched into 10 pairs according to Technique for Order Preference by Similarity to Ideal Solution score. As the unit of randomization, each pair of facilities is assigned at random to a control or an intervention group through coin flipping. Prescribed ranking information is publicly reported in the intervention group. The public materials include the posters of individuals and of facilities, the ranking lists of general practitioners, and brochures of patients, which are updated monthly. The intervention began on 13th November 2013 and lasted for one year. Specifically, participants are surveyed at five points in time (baseline, quarterly following the intervention) through questionnaires, interviews, and observations. These participants include an average of 600 patients, 300 general practitioners, 15 directors, and 6 health bureau administrators. The primary outcomes are the transparency mechanism model and the changes in medicine-prescribe. Subsequently, the modifications in the transparency mechanism constructs are evaluated. The outcomes are measured at the individual participant level, and the professional who analyzes the data is blind to the randomization status.

**Discussion:**

This study protocol outlines a design that aims to examine the transparency mechanism and to evaluate the effect of public reporting on prescription. The research design is significant in the field of public policy. Furthermore, this study intends to fill the gap of the investigation of the transparency mechanism and the evaluation of public reporting on prescription.

**Electronic supplementary material:**

The online version of this article (doi:10.1186/s12889-015-1454-6) contains supplementary material, which is available to authorized users.

## Background

### Transparency as a popular policy issue

Transparency has become one of the most popular topics and is a widespread normative doctrine for governance conduct [1,2]. The value of transparency has rarely been questioned now. Many governments and organizations, such as the US and the European Union, have designed public systems to reduce financial, health, and safety field risks, as well as to improve public services [3,4].

In the field of healthcare, the public reporting of the performance data obtained from providers has been widely used to improve the quality of care. Almost all US states have implemented numerous reporting programs for hospitals, and the Center for Medicare and Medicaid Services has initiated public disclosure programs for hospitals, manages care plans, nursing homes, and home health agencies [5]. In South Korea, a national insurance review agency has been releasing information regarding antibiotics use rates among healthcare organizations publicly since 2006 [6].

In 2009, the Chinese government emphasized the importance of increasing transparent regulation in the medical field, such as on the rational use of antibiotics and on prescription comments. This policy is highly significant in promoting transparency in medicine use, which is a most urgent necessity. Many local governments have attempted to develop such policies; however, few research results have been published thus far.

### Poor sound evidence for the medical public

Many studies that evaluate the effect of the public reporting of performance data on healthcare quality improvement report mixed results [7,8]. Hannan et al. [9] and Schneider and Epstein [10] of public reporting systems in the US have reduced cardiac surgery mortality and have motivated hospitals and health plans to improve the quality of care provided. However, critics have noted that the link of public reporting to improved outcomes lacks evidence. Jang [11] reported that the repeated release of cesarean section rates to the public did not significantly lower these rates. Furthermore, Bundorf [12] assessed a reporting program for Medicare health maintenance organizations and stated that public reporting does not significantly and positively affect quality. Moreover, few studies have examined the relationship between the public reporting of prescribed ranking information (PRPRI) and the rational use of medicines (RUM) [13].

In view of the methodology, few transparency studies have conducted cluster randomized controlled trials. A comparison group was set in Bundorf’s [12] study, but a new policy was implemented during the study period. Jang’s [11] study also generated a control group, but this study involved the analysis of secondary data. Furthermore, confounding was not matched. Hibbard [14] applied a quasi-experimental method and set a control group, but this group was not randomly selected. Therefore, either a high-quality experimental or a cluster randomized controlled trial design must be developed to determine whether the public reporting of information influences quality improvement.

### Few related theories for and empirical research on the transparency mechanism

Berwick, James, and Coye [15] indicated that public reporting can improve performance in two ways (selection and change). These aspects are interconnected by the motivation of a provider to maintain or to increase market share. Fung [16] proposed the transparency action cycle. According to the theory, “Effective public transparency systems trigger a virtuous chain of action and reaction. First, consumers, voters or other information users react to new facts by changing their perceptions and behavior. Second, manufacturers, political candidates or other information disclosers change their perceptions and behavior in response to users’ actions in order to improve their competitive advantage”. Both Berwick and Fung reported that public reporting should be advocated to improve the healthcare quality of providers by presenting information, selection, and change activities.

Little empirical research has been conducted on the transparency mechanism. Sherman et al. [17] evaluated the perceptions of surgeons at hospital-level and individual-level public reporting and identified specific barriers to their acceptance of this reporting. This study enhances understanding regarding the concerns of surgeons with regard to public reporting and promotes its widespread implementation. Hibbard, Stockard, and Tusler [18] indicated that patients who can perceive public reporting and the mechanism by which it can affect quality improvement accurately are more likely to be concerned with reputation than with market share.

### Research questions and significance

The Institute of Medicine defines healthcare transparency as the concept of making healthcare available to the public in a reliable and understandable manner. It mainly focuses on the reporting of information and processes [19]. In the current study, we emphasize only public reporting in the field of medicine use. Our research questions include the follows: “Is Fung’s theory applicable in the field of medicine use?” How does it work? If it does not work, why?

This research aims to fill a gap in the literature on the investigation of the transparency mechanism and the evaluation of public reporting on prescription by applying a cluster randomized controlled trial design. It intends to contribute significantly to both theory development and practical guidance. First, tools are developed to measure the key constructs of the transparency mechanism and to establish and test relationships among the constructs based on Fung’s theory in the field of medicine use. Second, a controlled randomized cluster trial is applied to evaluate the effect of PRPRI on RUM and to provide a reference to researchers and policy makers with respect to the effect of public reporting based on sound evidence.

### Objectives

The primary objective is to investigate the transparency mechanism in the field of medicine use based on Fung’s transparency action cycle and to test the robustness of this mechanism using five times’ panel data. The secondary objective is to evaluate the effect of PRPRI on prescription, namely, whether PRPRI can promote the RUM of a general practitioner (GP). All objectives are considered at the level of individual participants.

**Figure 1:**
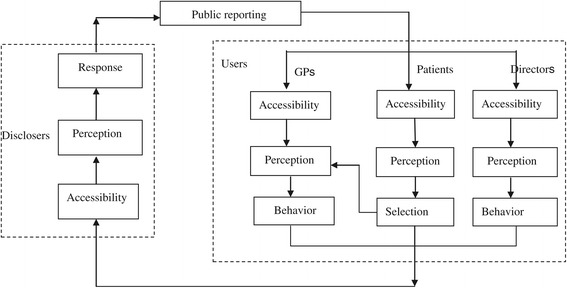
**Study framework of the transparency action cycle.**

The main hypotheses in this study contain the following points: (1) PRPRI can promote RUM; (2) PRPRI can affect information accessibility, perception, stress level, and the behavior intention of information users (including GPs, directors of primary-care facilities, and patients at these facilities, as well as their families); (3) the original information disclosers may in turn perceive the changes in the users and respond accordingly (these disclosers include research team and the local health bureau). Based on Fung’s transparency action cycle, all of these hypotheses comprise the framework of our research, which is illustrated in Figure 1.

## Methods

### Study design

This study employed a cluster randomized controlled trial that was designed to comply with the guidelines of the Consolidated Standards of Reporting Trials (CONSORT) [20]. An additional file shows this in more detail (see Additional file 1). The reasons for adopting cluster randomization include the following: (1) it prevents contamination because GPs and patients who come from one primary-care facility may either share or unintentionally transfer intervention effects from one group to another; (2) clustering at the level of the primary-care facility conveniently implements intervention and improves administrative efficiency. The clusters are the primary-care facilities in Q City, which served more than one billion outpatients and emergent patients, and assisted 30,000 inpatients on average in 2013.

Primary-care facilities were matched in pairs according to Technique for Order Preference by Similarity to Ideal Solution (TOPSIS) score. This score was generated as per nine indicators, including the service population, approval beds, number of physicians, number of outpatients, number of inpatients, revenues, payment for performance, and the time and number of RUM training courses. **First**, we calculated the TOPSIS score of all 22 primary-care facilities in Q City. Given the limited number of researchers and financial resources, the two facilities with the lowest TOPSIS score, were removed. **Second,** we arranged the 20 facilities in the order of descending TOPSIS score and marked them with numbers 1 to number 20. Thus, we can sequence the numbers for the 20 facilities. **Third,** we matched two consecutive primary-care facilities into one pair. For example, the facility labeled number 1 was matched with that set as number 2. A total of 10 matched pairs of facilities were generated. **Finally,** one facility from each matched pair was randomly allocated by coin flipping to the intervention group while the other was assigned to the control group.

**Figure 2:**
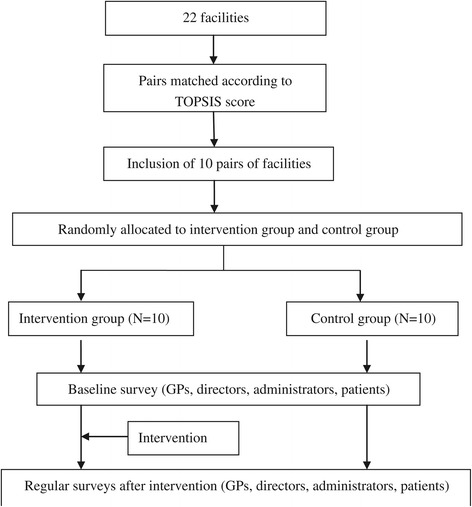
**Design of the matched-pair cluster randomized controlled trial.**

In the intervention group, the medicine-prescribing ranking information that ranks the personnel and the facilities was made public to GPs, directors, patients, and their families. In addition, patients were also provided with brochures that contain information regarding RUM and medicine-prescribing ranking information. The control group does not enforce such public reporting activities. The intervention lasted for one year, starting from November 2013. Survey data were collected at five points in time (baseline and quarterly after intervention) using questionnaires, interviews, and observation (Figure 2).

### Setting and participants

This study was conducted in Q City, which is a typical city in central Hubei Province. This city has 1,030,000 inhabitants and covers approximately 2004 km^2^. As per eligibility criteria for cluster level, the facility should be located in Q City; it must agree to participate in the trial; and it must have an excellent hospital information system (HIS).

The individual-level participants in this research included information users and disclosers. All GPs who work in a participating facility and have the license to prescribe medicine during our intervention period were eligible for the study. Patients were included only if they met the following criteria: they should have noted drug reimbursement, the price of the medicine, or other medical information; they must be able to understand our intervention materials and communicate with the investigators; and those in the intervention group should also have paid attention to the publicly reported prescribed ranking information. The families of the patients could be eligible if either the young or the elderly patients could not answer the questionnaires. The criteria for the eligibility of the directors of the facilities and of health bureau administrators for the study are as follows: they must be active participants, and they must be responsible for public reporting.

### Sample size

The research team completely enumerated all eligible GPs, and an average of 300 GPs were surveyed at each time point. We conducted purposive sampling to determine the interviewees. Moreover, sample sizes typically rely on the gold standard of “saturation”, which is the point at which no new information or theme is observed in the data [21]. It estimates the interviews of 15 directors and 6 administrators in advance at each time point.

This study adopted convenience sampling to investigate the patients. The sample size of patients is calculated as follows. **First,** we calculate the number of patients in an individually randomized trial (*n*_*1*_). According to Hibbard, Stockard, and Tusler [18], 63% of the patients in the intervention group and 40% of the patients in the control group patients are aware of public reporting in the medical healthcare field. We assumed that the probability estimates of the outcome indicators were *p1* = 0.63 and *p2* = 0.4 in our intervention and control groups, respectively. Given an alpha value of 5% and a beta value of 20%, we calculated *n*_*1*_ = 113 for each group based on Formula (1) [22]. **Second,** we computed the required sample size (*n*_*c*_) for a cluster randomized controlled trial based on individually randomized trial sample size using Formula (2), which was developed by Hemming et al. [23]. In our study, the number of clusters (*k*) was fixed at 10 per arm, and we assumed that the intra-cluster correlation coefficient (ρ) is 0.05. Therefore, we calculated 247 per arm as the required sample size for our cluster randomized trials. Supposing that the valid response rate is 95% based on our previous survey experience, we enlarged the sample size to 260 patients for each group. Finally, 26 patients from each facility should be investigated at each time point.

1 $$ {n}_1=\frac{\left({z}_{\upbeta}+{z}_{0.5\alpha}\right)2}{2*\left( \arcsin \sqrt{p1}- \arcsin \sqrt{\mathrm{p}2}\right)2} $$

2 $$ {n}_c=\frac{n_1k\left(1-\rho \right)}{k-{n}_1\rho } $$

### Randomization and blinding

The randomization sequence was set by coin flipping. For example, flip a coin and determines heads of the number one facility and then the number two facility is tails. We flipped the coin until all 20 facilities are marked as heads or tails. Therefore, the randomization sequence was established by flipping the coin 10 times prior to the beginning of program implementation. The facilities marked as heads were allocated to one group as intervention group and those marked with tails to another. The primary-care facilities were the unit of allocation, and the research team alone was aware of the allocation of all of the clusters. Blinding the research team was impossible because it plans and implements the intervention activities. Furthermore, participants were told only that they are participating in a trial, but they were not aware of their allocation. The professional who analyzed the data was blinded to the randomization status of the primary-care facilities until statistical analysis is completed.

### Recruitment

The primary-care facilities (clusters) are recruited via the local health bureau. The health bureau introduced the objectives and design of the cluster randomized controlled trial to these facilities and invited them to participate. All eligible participants (directors, administrators, patients, and all GPs in each facility) were recruited through word-of-mouth from members of the project team prior to each survey period. The participants were informed of the purpose of the trial and that the questionnaire data were confidential and would be used only for research purposes. They also received the information sheet and were given sufficient time to read it, to consider any implications, and to raise any questions to the investigators prior to deciding to participate.

### Intervention

The intervention aimed to publicly report the ranking of antibiotic prescription percentage, injection prescription percentage, and average drug cost per prescription at both facility and GP levels. The public materials included the posters of individual GPs and of facilities, the ranking lists of GPs, and the brochures of patients. We calculated the public materials based on the prescription data of the outpatients in the previous month and updated the public materials monthly. The ranking poster of the facilities pertained to cluster-level intervention. The poster and ranking list of the GPs and the brochures of patients pertained to individual participant-level intervention. The intervention was a multi-level intervention, but it targeted individual-level variables.

### Intervention materials

#### Ranking list of GPs

The ranking list contained the names of GPs and departments, antibiotic prescription percentage, injection prescription percentage, the average drug cost per prescription, the rankings in departments, and the star rankings among the 10 facilities. The ranking in departments was based on a comprehensive indicator calculated according to antibiotic prescription percentage, injection prescription percentage, and the average drug cost per prescription as determined by TOPSIS. The ranking results were presented in ascending order. The star rankings among the 10 facilities denoted the ranks of all GPs in the same departments of 10 facilities. The calculation method of these star rankings was similar to that for the rankings in departments. One-third of the highly ranked GPs were ranked with three stars, one-third of those ranked in the middle were ranked with two stars, and the remaining GPs were ranked with only one star. The higher the rank result, the more stars were accorded. The calculation method and rankings were interpreted at the bottom of the ranking list, in which high ranks and many stars represented the use of high-quality medicine. The ranking lists of GPs were printed in black on A4-size paper and were distributed to them monthly.

#### Bulletin board and the poster

The poster included the ranking posters of the GPs and facilities. Many information users are not medical professionals; hence, the posters were simplified based on the ranking list of the GPs. The ranking poster of the GPs included only the names of the GPs and departments, the rankings in departments, and the star rankings among the 10 facilities. The ranking posters of facilities included the names of the facilities and the rankings among the 10 facilities. The rankings of the facilities was based on a comprehensive indicator that was calculated based on the antibiotic prescription percentage, the injection prescription percentage, and the average drug cost per prescription at the facility level as determined by TOPSIS. The ranking results were sorted in ascending order. Both types of posters provided the interpretation of the calculation method and rankings at the bottom. The ranking posters of the GPs and facilities were color-printed on A3-size paper and were posted on the provided bulletin board measuring 0.8 m × 1.2 m.

#### Brochures of patients

The brochures of patients included the contents of both the facilities’ poster and the ranking list of GPs, as well as relevant knowledge regarding the concept of RUM, the harmful effect of irrational medicine use, and the significance of RUM promotion. All brochures were printed in color on A4-size paper.

### Intervention procedure

#### Preparation of intervention materials

First, outpatient prescription data from the 5th to the 10th of each month were retrieved from the HIS through the local health bureau starting from October 2013. Second, the RUM indicators for each GP and facility were calculated and then ranked based on TOPSIS. The RUM indicators used to monitor the prescription behavior in our intervention were adapted from those of the WHO/INRUM (International Network for the Rational Use of Drugs), which were widely used [24-26]. To ensure the accuracy of the calculation results, the ranking outcomes were cross-checked by two researchers. Third, the intervention materials were printed by a professional printing company.

#### Publicly reported intervention materials

The investigator posted the ranking posters and disseminated the ranking list to 10 intervention facilities and to each GP from the 10th to the 15th of each month. Simultaneously, our researchers distributed the brochures to the outpatients, inpatients, and their families. The rest of the brochures were placed in the publicity column, which was accessible to the patients. All of the intervention materials were provided to each facility director monthly in the intervention group. Throughout the entire intervention process, 120 copies of ranking posters of GPs, 120 ranking posters of the facilities, 1,800 ranking lists of GPs, and 1,000 of patient brochures were distributed.

#### Guaranteed measures of intervention

The health bureau of Q City issued documents to confirm that the posters should be protected and that they must not be destroyed or covered. The research team patrolled intervention facilities irregularly to verify the completeness and cleanliness of the posters. They also replaced the damaged posters.

### Data collection

#### Questionnaire survey

To develop the questionnaires for the transparency mechanism, we acquired references from previous studies. Items were adapted from other instruments and from expert opinions. Based on the literature review, a large pool of items was generated, and disputed items were removed as per several rounds of discussions among experts. Prior to collecting data in full, we conducted a pilot study to test the reliability and validity of the new measure and either removed or adjusted the items with low reliability. The questionnaire consisted of four constructs: information accessibility, perception, stress, and behavior intention for both GPs and patients. All items were measured with a five-point Likert scale. The survey also included demographic variables, such as age, sex, income, and education levels.

This study conducted five investigations on GPs (baseline, 3, 6, 9, and 12 months after intervention) and three investigations on patients (baseline, 3 and 12 months after intervention) for both the intervention and the control groups. Panel data from the two groups were used to test the robustness of the mechanism with the structural equation model (SEM).

#### Interview with key informants

We designed an interview outline and conducted four semi-structured interviews during the intervention period. The directors of primary-care facilities and health bureau administrators were our key interview objects because they communicated with and coordinated the research team and the facilities to ensure the smooth implementation of the intervention. We also interviewed key GPs that prescribed more than 200 prescriptions per month. The interview focused mainly on the transparency mechanism and its function conditions. Meanwhile, the general characteristics of the facilities, RUM promotion activities, such as commenting on prescriptions, RUM training, and the highest limit of antibiotic and injection prescription percentage, are also involved. The interview data were used to formulate and interpret the transparency mechanism using grounded theory method.

#### Observation survey

The research team observed the attitudes and behavior of directors and key GPs toward the intervention. The observation diary recorded the events (what), times (when), places (where), processes (how), and reasons (why). These records were as detailed as possible and were used to interpret the transparency mechanism.

#### Outpatient prescription data

The prescription data of all of the outpatients from the 20 facilities during the period of October 2013 to October 2014 were collected monthly. These data include patient IDs and medicine information, such as medicine name, dosage, and price. They were used to calculate the RUM indicators for public materials and to evaluate the effect of intervention.

### Quality assurance

The questionnaire survey was distributed according to standard operational protocol, and the investigators were trained accordingly. A face-to-face survey was conducted to emphasize timely explanations to respondents. We also incorporated mutual verification questions into the questionnaire. If the chosen items were contradictory, then the respondents were required to answer the questionnaire again. The double data entry method was used to input paper-based questionnaires. Inconsistencies were investigated by reviewing the paper-based version.

Notes were taken during then interviews. The researchers also recorded the interviews in full after obtaining the consent of the interviewees. Two researchers who were blind to the intervention group independently encoded the interview data. Disagreements were resolved via discussions with the other researchers.

Prior to the start of the investigations, the health bureau issued documents to highlight the importance of the study and to inform facilities and GPs to cooperate in responding to the survey. Additionally, patients and GPs were given small gifts, such as umbrellas, cups, and toothpaste, to improve cooperation and questionnaire response rate.

### Outcome measures and statistical analysis

The **primary outcomes** are the transparency mechanism model and the change in five RUM indicators before and after intervention. The **secondary outcomes** are the modifications in transparency mechanism constructs, including information accessibility, information perception, stress level, and behavior intention. Outcomes were measured at the individual participant level.

The multi-method design included both qualitative and quantitative methods, such as generalized estimating equations (GEE) and grounded theory. Quantitative analysis was conducted using STATA (version 12.0). Furthermore, the software NVivo 8.0 was employed to manage and index transcriptions into a coding system. The specific analysis indicators and the statistical analysis are listed in Table 1.

**Table 1:** **Outcomes and statistical analysis of the study**

**Outcome measures**			**Statistical analysis**
**Outcomes**	**Indicators or constructs**	**Description**	
RUM	Antibiotic prescription percentage	Calculated by dividing the number of antibiotic prescriptions to patients by the total number of prescriptions in a certain period of time. This value is then multiplied by 100.	**Propensity Score Matching** (**PSM**): This method reduces the influence of bias and confounding variables and reasonably assesses the intervention and control groups;
	Injection prescription percentage	Calculated by dividing the number of injection prescriptions to patients by the total number of prescriptions in a certain period of time. This value is then multiplied by 100.	**Difference-in-difference (DID)**: The variations in an index of the two groups before and after intervention are calculated to reflect the net effect of intervention. The differences lie in the cluster-level summaries of the two groups.
	Average drug cost per prescription	Calculated by dividing the total cost of all drugs prescribed by the number of prescriptions in a certain period of time.	**Generalized estimating equations (GEE)**: For multivariate outcome analyses, we used GEE to assess the effect of PRPRI intervention with repeated measures at the individual participant level. For continuous dependent variables, we used the GEE model with a normal apply and an identity link function. For dichotomous dependent variables, we employ the GEE model with a binomial distribution and a logit link function.
	Prescription percentage of drugs listed in the essential drug list or formulary	Calculated by dividing the number of prescribed products listed on the essential drugs list or local formulary (or of those that are equivalent to drugs on the list) by the total number of products prescribed in a certain period of time. This value is then multiplied by 100.	
	Percentage of prescription with duplicate or more prescribed antibiotics	Calculated through dividing the number of patient prescriptions during the time a duplicate or more antibiotics were prescribed by the total number of prescriptions in a certain period of time, multiplied by 100.	
Robustness of the transparent mechanism	Information accessibility	Accessibility score	**Factor analysis**: This method evaluates the reliability and validity of the questionnaire.
	Information perception	Perception score	**Structural equation modeling (SEM)**: This technique builds the inner link of each construct of the transparent mechanism and tests the robustness of the mechanism by applying the questionnaire data from two groups four times.
	Stress level	Stress score	
	Behavior intention	Behavior intention score	
Development of the transparent mechanism	Constructs, such as accessibility, perception, and behavior intention	The code of information accessibility, perception, behavior, and other related influence factors.	**Grounded theory**: The qualitative data were analyzed through a thematic framework. Codes were then developed based on the viewpoints that were derived from these data.

### Ethics

This study was approved by the Ethics Committee of Tongji Medical College, Huazhong University of Science and Technology (NO: IORG 0003571). It was also permitted by the local health bureau. Patient names and other confidential data included in outpatient prescription information were secured according to medical confidentiality rules.

## Discussions

This paper describes the protocol of a matched-pair cluster randomized controlled trial that aims to explore and test the robustness of the transparency mechanism, as well as to evaluate the effect of public reporting on prescription in primary-care facilities. Although the transparency mechanism and effect have been examined and assessed in previous studies [7,17,18], our trial has some unique features.

First, the large-scale, one-year trial investigates both information users and disclosures quarterly through questionnaires, interviews, and observations methods. Each investigation surveys 300 GPs and 600 patients. In addition, 700,000 prescriptions are retrieved during our study. Moreover, the approaches of qualitative and quantitative methods were mixed. For example, SEM was used to establish and to test the robustness of the transparency mechanism with five times’ panel data. Grounded theory was applied to analyze the interview materials as an important supplemental evidence for transparency mechanism research. Meanwhile, the GEE and DID method was employed to study the outpatient prescription data retrieved from HIS and evaluate the effect of intervention.

Second, another distinguishing feature of the design is its focus on primary-care facilities. Previous studies usually research the public reporting issues in hospitals [9-13]. Primary-care facilities are increasingly important worldwide as gatekeepers of good health [27]. Elucidating the mechanism and effect of transparency in primary-care facilities can not only improve healthcare services for 1.4 billion Chinese but also provides references for other countries.

The study in Q City provides strong evidence for the examination of transparency mechanism and for the evaluation of the effect of public reporting on prescription. Furthermore, this study fills the gap of the investigation of transparency mechanism and the evaluation of PRPRI on prescription. Thus, it can contribute significantly to both theory development and practice. With respect to theoretical research, this study identifies and develops measurements of the key constructs and variables of the transparency mechanism. It also establishes relationships among the constructs in the field of medicine use. In terms of practical guidance, this research enhances understanding regarding the concerns of stakeholders and the behavioral change in public reporting. Hence, this study significantly facilitates RUM and promotes the widespread implementation of public reporting.

Nonetheless, our design also has some limitations. First, the grouping process according to TOPSIS score considers only the key influence factors of RUM and does not take into account the prescription factor at the baseline levels of the 20 primary-care facilities. Therefore, the DID analysis method was used in the study to remedy the deficiency of the design [28]. Second, the Hawthorne effect may be induced during our study, especially during the interviews. We will improve the communication between the researchers and the participants to reduce this effect as much as possible [29]. Finally, the intervention lasts for only one year, but the observation of some effects may require long-term follow-up surveys. Therefore, we may have underestimated the detected intervention effect.

## Additional file

Additional file 1:
**CONSORT 2010 Cluster RCT Checklist.**

## Abbreviations

CONSORT: Consolidated standards of reporting trials
DID: Difference-in-difference
GEE: Generalized estimating equations
GP: General practitioner
HIS: Hospital information system
INRUM: International Network for the Rational Use of Drugs
PRPRI: Public reporting of prescribing ranking information
PSM: Propensity score matching
RUM: Rational use of medicines
SEM: Structural equation modeling
TOPSIS: Technique for Order Preference by Similarity to Ideal Solution
WHO: World Health Organization
